# Transcriptomic Analysis Reveals Abnormal Expression of Prion Disease Gene Pathway in Brains from Patients with Autism Spectrum Disorders

**DOI:** 10.3390/brainsci10040200

**Published:** 2020-03-29

**Authors:** Salvo Danilo Lombardo, Giuseppe Battaglia, Maria Cristina Petralia, Katia Mangano, Maria Sofia Basile, Valeria Bruno, Paolo Fagone, Rita Bella, Ferdinando Nicoletti, Eugenio Cavalli

**Affiliations:** 1 Department of Biomedical and Biotechnological Sciences, University of Catania, 95123 Catania, Italy; salvo.lombardo.sdl@gmail.com (S.D.L.); kmangano@unict.it (K.M.); sofiabasile@hotmail.it (M.S.B.); ferdinic@unict.it (F.N.); eugeniocavalli9@hotmail.it (E.C.); 2 Department of Physiology and Pharmacology, University Sapienza, Piazzale A. Moro, 5, 00185 Roma, Italy; giuseppe.battaglia@uniroma1.it (G.B.); valeria.bruno@uniroma1.it (V.B.); 3 IRCCS Neuromed, Località Camerelle, 86077 Pozzilli, Italy; 4 Department of Educational Sciences, University of Catania, 95124 Catania, Italy; m.cristinapetralia@gmail.com; 5 Department of Medical Sciences, Surgery and Advanced Technologies, University of Catania, 95123 Catania, Italy; rbella@unict.it

**Keywords:** autism, infection, prion, meta-analysis

## Abstract

The role of infections in the pathogenesis of autism spectrum disorder (ASD) is still controversial. In this study, we aimed to evaluate markers of infections and immune activation in ASD by performing a meta-analysis of publicly available whole-genome transcriptomic datasets of brain samples from autistic patients and otherwise normal people. Among the differentially expressed genes, no significant enrichment was observed for infectious diseases previously associated with ASD, including herpes simplex virus-1 (HSV-1), cytomegalovirus and Epstein–Barr virus in brain samples, nor was it found in peripheral blood from ASD patients. Interestingly, a significant number of genes belonging to the “prion diseases” pathway were found to be modulated in our ASD brain meta-analysis. Overall, our data do not support an association between infection and ASD. However, the data do provide support for the involvement of pathways related to other neurodegenerative diseases and give input to uncover novel pathogenetic mechanisms underlying ASD.

## 1. Introduction

Autism spectrum disorder (ASD) is a heterogeneous group of neurodevelopmental disorders defined by significantly abnormal social interaction, impaired communication and language abilities, and a narrow pattern of interests [1]. It is estimated that the prevalence of ASD is 1%–2% in the general population with an average male-to-female ratio of 5:1 [2]. However, only about 10% of patients with a diagnosis of ASD have a defined etiology (so-called syndromic autism, secondary to Fragile X syndrome, neurofibromatosis and exposure to thalidomide) [3], while 90% of ASD cases are considered idiopathic.

Many authors have hypothesized a connection between genetic and epigenetic factors in ASD etiopathogenesis. In particular, infections have been suggested as a potential trigger of the disease [4,5,6]. In line with this, altered cellular immunity and an altered cytotoxic function of natural killer (NK) cells have been reported in ASD patients [7,8,9]. It has also been shown that fungal mycotoxins, such as deoxynivalenol in urine and Ochratoxin A in serum, are increased in autistic children [10,11]. Finally, expression of immune response genes has been described in cortical tissues from older ASD subjects [12,13].

In the present study, we investigated the expression levels of transcriptional markers of infections and immune activation in brain and blood samples from autistic patients by performing a meta-analysis of publicly available whole-genome expression datasets. The analysis of the data suggests common transcriptional features between ASD and prion-related diseases but does not support the role of infectious disease in the etiopathogenesis of ASD.

## 2. Materials and Methods

### 2.1. Data Collection and Metanalysis

The NCBI (National Center for Biotechnology Information) Gene Expression Omnibus (GEO) database (http://www.ncbi.nlm.nih.gov/geo/) was used to identify microarray datasets comparing the transcriptomic profiles of healthy donors and ASD patients. The GEO database was manually searched using the terms “autism” and “autistic disorder”. The collected datasets were further selected if they met the following inclusion criteria: (a) whole-genome transcriptomic profiling; (b) brain or blood samples; (c) consisted of one cohort of ASD patients and another cohort of healthy people; and (d) species of origin was “*Homo sapiens*”. Finally, five datasets were included in the meta-analysis of brain samples: GSE28521, GSE38322, GSE62098, GSE64018 and GSE102741, while three datasets were used for the meta-analysis of blood samples: GSE6575, GSE42133 and GSE18123. When a dataset included more than one tissue type, data from each tissue type were processed as a separate dataset. The datasets were uploaded to NetworkAnalyst 3.0 software (Ste. Anne de Bellevue, Quebec, Canada). Data were auto-scaled, and an integrity check was performed prior to the meta-analysis stage. Batch effects were corrected using the “ComBat” function. A random effects model of effect size (ES) measure was used to integrate gene expression patterns from the three datasets. The random effects model presumes that different studies present substantial diversity and evaluates between-study variance as well as within-study sampling error. Genes with a False Discovery Rate (FDR) < 0.05 were identified as differentially expressed genes (DEGs) and selected for further analysis. The characteristics of the samples in the datasets used are described in Table 1.

### 2.2. Pathway Selection and Gene Intersection

Pathway enrichment analysis was performed using the Kyoto Encyclopedia of Genes and Genomes (KEGG) database (https://www.genome.jp/kegg/) implemented in the Enrichr (http://amp.pharm.mssm.edu/Enrichr) web-based utility [24]. Higher-level biological functions are represented by networks of molecular interactions, reactions and relations that are integrated in the pathways from the KEGG database. KEGG integrates the current knowledge on molecular interaction networks and uses a knowledge-based approach for network prediction that aims to predict, given a complete set of genes in the genome, the protein interaction networks that are responsible for various cellular processes [25]. Enrichr computes the *p* value using the Fisher exact test. The adjusted *p* value is calculated using the Benjamini–Hochberg method for correction for multiple hypotheses testing. The z-score is computed using a modification to the Fisher exact test and assesses the deviation from the expected rank. Finally, the combined score is calculated using the *p* value and the z-score (Combined Score = ln(*p* value) × z-score).

### 2.3. Machine Learning Prediction and Network Construction

The webtool “ASD Genome-wide predictions of autism-associated genes” was used to evaluate the probability value of association between the selected gene and ASD. This webtool is based on a machine learning approach that, using a Bayesian method, allows the user to predict the role of candidate genes [26]. Briefly, Krishnan et al. developed an evidence-weighted, network-based machine-learning method that uses this brain-specific network to systematically discover new candidate ASD risk genes across the genome. The brain-specific network was constructed using a Bayesian method that extracts and integrates brain-specific functional signals from a gene-interaction network model containing predicted functional relationships for all pairs within 25,825 genes in the human genome. In order to produce a comprehensive, robust, genome-wide ranked list of autism candidate genes, Krishnan et al. first curated 594 genes linked with autism from publicly available databases and based on the strength of evidence of association with ASD. Next, an evidence-weighted support vector machine classifier, using the connectivity of genes to all the genes in the human brain-specific network, was employed to identify novel ASD candidates, defined as those genes whose interaction features in the network most closely resemble those of known ASD-related genes [26].

### 2.4. Statistical Analysis

For the meta-analysis, a random-effect model of effect size measure was used to integrate gene expression patterns from the selected datasets. Genes with an adjusted *p* value (FDR, q-value) < 0.05 were identified as DEGs and selected for further analysis. Pathway enrichment analysis was performed using the online server Enrichr (http://amp.pharm.mssm.edu/Enrichr) [24]. For all the analyses, an adjusted *p* value ≤ 0.05 was considered as the statistical significance threshold.

## 3. Results

### 3.1. Identification of an ASD Brain Transcriptomic Profile

Five GEO whole-genome transcriptomic datasets were identified (see Table 1) and used in the following analysis. These datasets included 84 brain samples from ASD patients (*n* = 55 unique patients) and 109 brain samples from otherwise normal people (*n* = 81 unique subjects). The meta-analysis identified 516 DEGs: 218 upregulated and 298 downregulated. The most enriched pathways were represented by “Synaptic vesicle cycle”, “Huntington’s disease” and “Sphingolipid signaling pathway” (Table 2).

**Figure 1:**
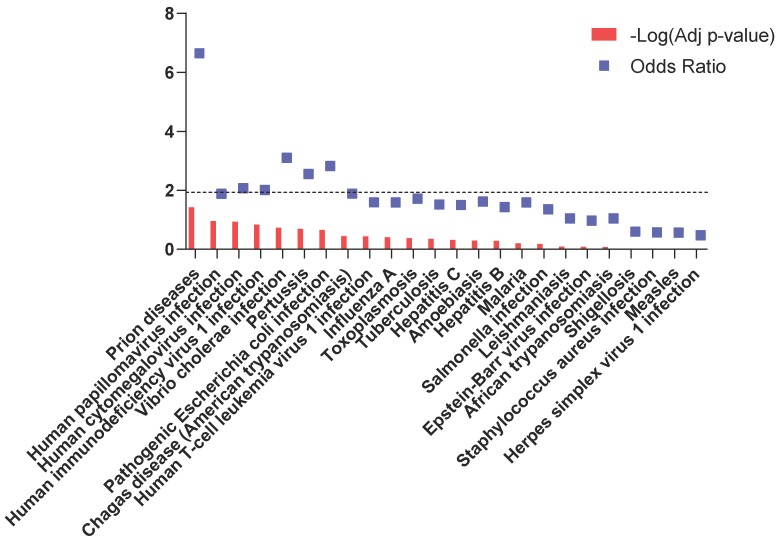
Infection-related pathways enriched in brain samples from ASD patients. Dotted line indicates the threshold of significance.

**Figure 2:**
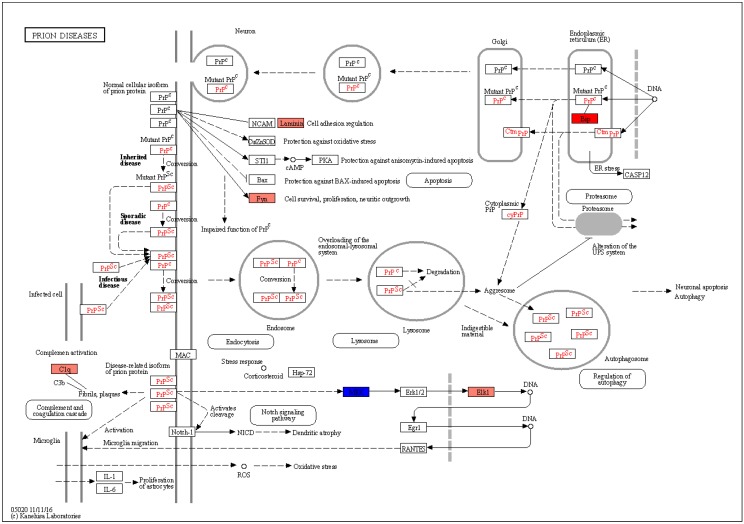
“Prion diseases pathway” from the Kyoto Encyclopedia of Genes and Genomes (KEGG) database with genes significantly modulated in brain samples from ASD patients that have been color-coded from blue (downregulated) to red (upregulated).

Figure 1 shows the results from the enrichment analysis for infectious-related pathways enlisted in the KEGG database. No significant enrichment was observed among the DEGs with the exception of the “prion diseases” pathway (q = 0.038) (Figure 1 and Figure 2; Supplementary File 1). In particular, in the “prion diseases” pathway, our analysis identified significantly higher levels of Complement Component 1, q Subcomponent, B Chain (C1QB), Heat Shock Protein Family A Member 5 (HSPA5), Proto-Oncogene Tyrosine-Protein kinase Fyn (FYN), Laminin Subunit Gamma 1 (LAMC1) and ETS Like-1 Protein (ELK1) and significantly lower levels of Mitogen-Activated Protein Kinase 1 (MAP2K1) (Figure 2).

**Figure 3:**
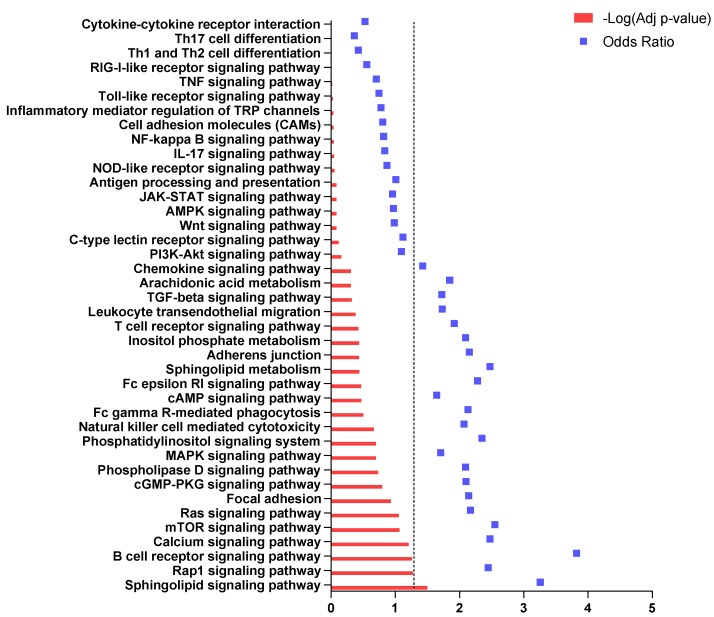
Immune-related pathways enriched in brain samples from ASD patients. Dotted line indicates the threshold of significance.

We next wanted to evaluate the enrichment of immune-related processes among the ASD brain DEGs. As shown in Figure 3, only the “Sphingolipid signaling pathway” was significantly enriched, encompassing four downregulated DEGs (MAP2K1, Protein Kinase C Beta (PRKCB), Sphingosine Kinase 2 (SPHK2), Ras-Related C3 Botulinum Toxin Substrate 3 (RAC3)) and six upregulated DEGs (G Protein Subunit Alpha I3 (GNAI3), Sphingosine-1-phosphate receptor 1 (S1PR1), FYN, Rapidly Accelerated Fibrosarcoma 1 (RAF1), TNF Receptor Superfamily Member 1A (TNFRSF1A), G Protein Subunit Alpha I2 (GNAI2)).

### 3.2. Machine Learning Prediction

**Figure 4:**
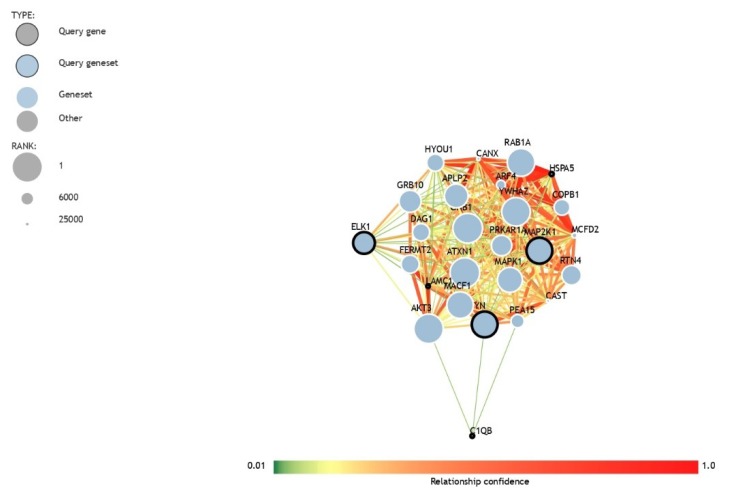
Network constructed using the differentially expressed genes in the ASD brain belonging to the “prion diseases” pathway using a minimum confidence score of 0.04 and a maximum of 20 interacting genes.

The brain autism DEGs belonging to the “prion diseases” pathway from the Kyoto Encyclopedia of Genes and Genomes (KEGG) were investigated for their potential role in ASD using a network machine learning approach implemented in the “ASD Genome-wide predictions of autism-associated genes” web-tool (http://asd.princeton.edu/). The network constructed using the brain ASD DEGs belonging to the “prion diseases” pathway is presented as Figure 4. Among the input genes, the only one significantly associated with ASD is FYN, with an estimated probability of 0.665 and a q-value = 0.0256. Table 3 shows the genes mostly interacting with the input genes, ordered by the edge score. The prioritization and prediction of ranking is based on the network-based approach developed by Krishnan et al. [26]. Among the top-ranking ASD genes associated with the DEGs belonging to the “prion diseases” pathway, Mesencephalic Astrocyte Derived Neurotrophic Factor (MANF), Heat Shock Protein 90 Beta Family Member 1 (HSP90B1) and Mitogen-Activated Protein Kinase 1 (MAPK1) showed edge scores of 0.791, 0.79 and 0.789 with HSPA5, HSPA5 and MAP2K1, respectively (Table 3). The top-ranking ASD gene interacting with the DEGs belonging to the “prion diseases” pathway is Ataxin 1 (ATXN1), with a rank position of 5, a probability value of association with ASD of 0.808 and a q-value = 0.0186. ATXN1 was the most connected gene to FYN (edge score 0.705) (Table 4). None of the predicted top 10 genes are present in the Genome-Wide Association Study (GWAS) Catalog 2019.

### 3.3. Identification of an ASD Blood Transcriptomic Profile

Three GEO whole-genome transcriptomic datasets, GSE6575, GSE42133 and GSE18123, were identified, as indicated in Table 1, for the following analysis. These datasets included blood samples from 157 ASD patients and blood samples from 101 otherwise normal people. The meta-analysis identified only 24 DEGs: 19 upregulated and 5 downregulated. As shown in Table 5, no significant enrichment for any KEGG pathway was detected (Table 5).

## 4. Discussion

According to the current DSM-5 criteria, two requirements are needed to obtain an ASD diagnosis: (1) persistent deficits in social communication and social interaction across multiple contexts, and (2) restricted, repetitive patterns of behavior, interests or activities [1]. Although ASD has a complex multifactorial etiology, twin studies have proven a strong genetic contribution, with a concordance rate of autistic disorders in monozygotic twins of 70%–90% and in dizygotic twins of 30% [27,28].

However, the complexity of the disease requires omics approaches to integrate and extrapolate more information. Genome-wide association studies, candidate gene studies and microarray experiments of differential gene expression have been largely used in autism. These studies produce extensive and information-rich data that represent a snapshot of all genetic and/or molecular events occurring in a diseased cell at one particular point in time and can be used to generate hypotheses. The use of whole-genome expression databases has been largely exploited by our group and others [29,30,31,32,33] for the characterization of the etiopathogenesis of a variety of diseases (e.g., autoimmune diseases [34,35,36,37,38,39,40,41,42] and cancer [36,43,44]) and has allowed researchers to characterize pathogenic pathways [45,46,47,48] and potential novel therapeutic targets [49,50,51,52,53,54,55,56,57].

Many authors have suggested that the role of infection during pregnancy or in the first phases of life could trigger the immune system to alter normal neurodevelopment, causing neuronal damage [8,58,59]. In particular, the role of the Herpesviridae family has been largely investigated. For instance, cytomegalovirus (CMV) can directly damage key structures in the developing brain when contracted during pregnancy [60], and indeed, in vitro studies have shown that CMV infection can inhibit neuronal differentiation and induce apoptosis in neural precursor cells [61,62]. Also, other infectious diseases such as influenza A [58], toxoplasmosis [63,64] and measles [6,65] are suspected to be related to ASD.

However, the role of infections in the pathogenesis of autism is still highly debated. The levels of D-arabinitol, a marker of candidiasis fungal infection, as well as of a phenylalanine metabolite of *Clostridia* species, the 3-(3-hydroxyphenyl)-3-hydroxypropionic acid, are increased in the urine of autistic children [66,67]. Accumulating evidence also suggests that latent chronic toxoplasmosis plays a role in the triggering and development of many psychiatric and neurological disorders, including ASD [68]. On the other hand, other studies have not shown a significant prevalence of infections in ASD [5,69,70,71]. The aim of our analysis was to evaluate, by performing a meta-analysis of available whole-genome transcriptomic datasets, whether infection alone or infection and immune activation processes could be detected in the brains or peripheral blood of autistic patients. To our knowledge, this is the largest meta-analysis of both ASD brain samples and leukocytes to date.

In our study, no significant enrichment for infection-related pathways, including Epstein-Barr virus (EBV), CMV, HSV-1, measles, influenza A and toxoplasmosis, was found among the DEGs identified in the meta-analyses. On the other hand, a significant enrichment of the “prion diseases” pathway was observed. However, it should be pointed out that, with the present data, it is currently not possible to identify ASD as a prion-related disease, but it is possible to describe common biomolecular pathways underlying ASD pathogenesis. Indeed, prion infection is known to affect microglial sensing and homeostasis ability and to reduce microglial phagocytosis of aberrant proteins, including PrPsc (scrapie isoform of the prion protein) and apoptotic debris or cells, despite production of proinflammatory mediators. Furthermore, the effects of PrPsc on microglia appear to be mediated by Toll-like Receptors (TLRs) in a Src-like kinase-dependent manner (reviewed in [72]). So, it may not be surprising to find that prion pathways are modified in the brains of ASD patients, as it may reflect prior inflammatory processes, having modified microglia.

In the present paper, we have combined transcriptomic meta-analysis, pathway enrichment and machine learning prediction in order to prioritize genes of interest with potential pivotal pathogenetic effects in autism. Computational methods have been largely used to investigate the etiopathogenesis of polygenic and idiopathic disorders. Functional interaction networks that integrate gene interaction data can be exploited to identify which genes are most strongly implicated in a disorder. Given a list of genes that are altered in a disease, we can apply methods to identify genes that are near the input genes within a functional interaction network that rely on the connections among genes in a functional interaction network. The major limitation of this kind of approach is that it relies on the methods of selection by which functional terms are included in the network-based prediction. Hence, the better tailored this set of genes is to the disease of interest, the higher reliability we have in the final predictions. The use of the machine learning prediction tool developed by Krishnan and colleagues [26] allows us to evaluate the probability value of association between the selected gene and ASD in the context of the human brain-specific network. With this approach, we likely arrive at a robust set of candidates that are relatively unbiased by previously published works. The final output of this strategy, i.e., a ranked list of candidate genes, is easy to interpret and provides a limited set of hypotheses to test in further investigations. However, while we cannot definitively identify the causal gene or genes, it does provide a much-reduced set of candidates to investigate. In particular, a role for tyrosine kinase Fyn is proposed. Fyn has been described as expressed in the mouse hippocampus, amygdala and cerebellum [73,74]. Mutations of Fyn in mice lead to alteration in the architecture of the hippocampus [75] with consequent impairment in learning and in the amygdala long-term plasticity [73]. Fyn regulates the focal adhesion kinase (FAK), which is required for normal neuronal development [73,76].

In our analysis, Fyn was strongly correlated with ATXN1, a DNA-binding protein that forms a transcriptional repressor complex with capicua (CIC). It has been previously described that the deletion in chromosome 6p22.3-p24.3, which harbors ATXN1, is associated with developmental delay and ASD [77,78]. Moreover, alteration of the ATXN1-CIC complex determines a spectrum of neurobehavioral phenotypes, including intellectual disability, attention deficit/hyperactivity disorder (ADHD) and autism spectrum disorder [79].

Finally, we need to address some important limitations to our study. First, the number of available gene expression datasets of brain samples derived from ASD patients is limited, and the number of samples included in each dataset is often negligible. Second, the meta-analysis here performed encompasses different brain regions (temporal, occipital and frontal cortex, as well as corpus callosum and cerebellum). These facts undermine the statistical power of the differential expression analysis and impede patients’ stratification, in terms of clinical phenotype, which is advisable given the heterogeneity of ASD. It is likely that different subgroups of patients may have peculiar brain transcriptomic patterns. Moreover, gene expression analysis is not enough to determine whether particular biological processes are activated or not, limiting the reliability of the conclusions that can be drawn. Hence, more population- and molecular-based studies are warranted to confirm or negate hypotheses.

Characterizing molecular pathways underlying ASD represents a crucial step for personalized medicine where comprehensive phenotyping of individual patients could be available, providing novel tailored treatment options. The data from this study suggest that infections may not necessarily be responsible for ASD development. However, since some genes involved in the infectious processes can interact with other key genes in autism, infections may likely act as co-factors, possibly causing worse clinical presentations. Future studies are necessary to validate these findings and prove if these genes can be used as biomarkers or even as eventual therapeutic targets. Finally, we have to point out that the present analysis cannot evaluate the potential role of infections in the prenatal period or contracted in the early stage of life.

## 5. Conclusions

In this paper, we investigated the relationship between infections and autism, proving that they should not be considered as etiological factors but probably as co-factors. We analyzed the gene expression profiles of brain and blood from autistic patients and compared them with the genes involved in the most frequent infectious diseases associated with pregnancy and suspected to be related to ASD. Our analysis does not show any statistically significant associations between ASD and previously studied infectious agents. However, it does show a statistical association between prion disease and autism. Finally, based on a Bayesian machine learning approach, we predicted that new genes may be associated with ASD and possibly, after validation, used as markers or therapeutic targets.

## Figures and Tables

**Table 1:** Characteristics of the datasets used in the meta-analyses.

Dataset ID	Tissue	Samples	Platform	Reference
GSE28521	Temporal cortex	*n* = 13 ASD *n* = 13 HD *	Illumina HumanRef-8 v3.0 Expression BeadChip	[14]
	Frontal cortex	*n* = 16 ASD *n* = 16 HD *		
	Cerebellum	*n* = 10 ASD *n* = 11 HD *		
GSE38322	Occipital cortex (BA19)	*n* = 6 ASD *n* = 4 HD *	Illumina HumanHT-12 V4.0 Expression BeadChip	[15] [16]
	Cerebellum	*n* = 8 ASD *n* = 8 HD *		
GSE62098	Corpus callosum	*n* = 6 ASD *n* = 6 HD *	Illumina HiSeq 2000 (*Homo sapiens*)	[17]
GSE64018	Superior temporal gyrus	*n* = 12 ASD *n* = 12 HD *	Illumina HiSeq 2000 (*Homo sapiens*)	[18]
GSE102741	Dorsolateral prefrontal cortex	*n* = 13 ASD *n* = 39 HD *	Illumina HiSeq 2000 (*Homo sapiens*)	[19]
GSE6575	Whole blood	*n* = 35 ASD *n* = 12 HD *	Affymetrix Human Genome U133 Plus 2.0 Array	[20]
GSE42133	Leukocytes	*n* = 91 ASD *n* = 56 HD *	Illumina HumanHT-12 V4.0 Expression BeadChip	[21] [22]
GSE18123	Whole blood	*n* = 31 ASD *n* = 33 HD *	Affymetrix Human Genome U133 Plus 2.0 Array	[23]

* HD: Healthy donors.

**Table 2:** Top 10 enriched KEGG pathways in brain samples from ASD patients.

Term	*p* Value	Adj. *p*-Value	Odds Ratio	Combined Score
Synaptic vesicle cycle	8.95E-04	0.030642	3.975353	27.90006
Huntington’s disease	5.06E-04	0.031192	2.811584	21.33503
Sphingolipid signaling pathway	0.001034	0.031855	3.257117	22.38962
Thyroid hormone signaling pathway	8.49E-04	0.03269	3.341353	23.62788
Parkinson’s disease	3.20E-04	0.032842	3.275467	26.35943
Gap junction	4.43E-04	0.034103	3.964059	30.61119
VEGF signaling pathway	7.81E-04	0.034369	4.598607	32.90204
Prion diseases	2.46E-04	0.037893	6.644518	55.21555
Valine, leucine and isoleucine degradation	0.001402	0.03926	4.844961	31.83014
Lysine degradation	7.81E-04	0.040097	4.598607	32.90204

**Table 3:** Top 10 genes interacting with ASD brain DEGs belonging to the “prion diseases” pathway.

Query Gene	Gene	Gene Description	Edge Score
HSPA5	MANF	mesencephalic astrocyte-derived neurotrophic factor	0.791
HSPA5	HSP90B1	heat shock protein 90kDa beta (Grp94), member 1	0.79
MAP2K1	MAPK1	mitogen-activated protein kinase 1	0.789
HSPA5	RAB1A	RAB1A, member RAS oncogene family	0.761
MAP2K1	PGK1	phosphoglycerate kinase 1	0.728
MAP2K1	YWHAZ	tyrosine 3-monooxygenase/tryptophan 5-monooxygenase activation protein, zeta polypeptide	0.706
FYN	ATXN1	ataxin 1	0.705
HSPA5	ARF4	ADP-ribosylation factor 4	0.702
HSPA5	HERPUD1	homocysteine-inducible, endoplasmic reticulum stress-inducible, ubiquitin-like domain member 1	0.69
LAMC1	AKT3	v-akt murine thymoma viral oncogene homolog 3	0.669

**Table 4:** Top 10 ranking ASD genes interacting with brain DEGs belonging to the “prion diseases” pathway.

Gene	Description	Avg. Edge Score to Query	Rank	Probability of ASD Association	*p*-Value	q-Value
ATXN1	ataxin 1	0.216	5	0.828	0.002	0.0186
GNB1	guanine nucleotide binding protein (G protein), beta polypeptide 1	0.231	24	0.811	0.001	0.0113
AKT3	v-akt murine thymoma viral oncogene homolog 3	0.205	75	0.722	0.001	0.0113
YWHAB	tyrosine 3-monooxygenase/tryptophan 5-monooxygenase activation protein, beta polypeptide	0.176	107	0.71	0.006	0.0438
YWHAZ	tyrosine 3-monooxygenase/tryptophan 5-monooxygenase activation protein, zeta polypeptide	0.212	269	0.697	0.08	0.3199
RAB1A	RAB1A, member RAS oncogene family	0.257	453	0.696	0.066	0.2786
MACF1	microtubule-actin crosslinking factor 1	0.205	715	0.667	0.005	0.0381
PPIB	peptidylprolyl isomerase B (cyclophilin B)	0.152	753	0.666	0.221	0.6426
BHLHE40	basic helix-loop-helix family, member e40	0.187	1054	0.663	0.149	0.4976
MAPK1	mitogen-activated protein kinase 1	0.234	1128	0.661	0.133	0.4605

**Table 5:** Top 10 enriched KEGG pathways in blood from ASD patients.

Term	*p*-Value	Adjusted *p*-Value	Odds Ratio	Combined Score
Autophagy	0.01023	0.450115	13.02083	59.6672
Osteoclast differentiation	0.010077	0.517306	13.12336	60.33413
cGMP-PKG signaling pathway	0.016766	0.573785	10.04016	41.04796
Tuberculosis	0.019321	0.595073	9.310987	36.74661
Oocyte meiosis	0.009775	0.60217	13.33333	61.70503
Cellular senescence	0.015641	0.602172	10.41667	43.31114
AMPK signaling pathway	0.009039	0.695998	13.88889	65.36409
Thermogenesis	0.031025	0.73505	7.215007	25.05748
Regulation of actin cytoskeleton	0.026947	0.754518	7.788162	28.14549
Insulin resistance	0.007379	0.757574	15.4321	75.75804

## Supplementary Materials

The following are available online at https://www.mdpi.com/2076-3425/10/4/200/s1: File 1: Prion diseases pathway.
